# Understanding Error Patterns: An Analysis of Alignment Errors in Rigid 3D Body Scans

**DOI:** 10.3390/jimaging9120255

**Published:** 2023-11-21

**Authors:** Julian Meißner, Michael Kisiel, Nagarajan M. Thoppey, Michael M. Morlock, Sebastian Bannwarth

**Affiliations:** 1 BSN Medical GmbH, Schützenstraße 1-3, 22761 Hamburg, Germany; 2 Institute of Biomechanics, Hamburg University of Technology, 21073 Hamburg, Germany

**Keywords:** 3D body scanning, accuracy, misalignment, error pattern, body shape

## Abstract

Three-dimensional body scanners are attracting increasing interest in various application areas. To evaluate their accuracy, their 3D point clouds must be compared to a reference system by using a reference object. Since different scanning systems use different coordinate systems, an alignment is required for their evaluation. However, this process can result in translational and rotational misalignment. To understand the effects of alignment errors on the accuracy of measured circumferences of the human lower body, such misalignment is simulated in this paper and the resulting characteristic error patterns are analyzed. The results show that the total error consists of two components, namely translational and tilt. Linear correlations were found between the translational error (R^2^ = 0.90, … 0.97) and the change in circumferences as well as between the tilt error (R^2^ = 0.55, … 0.78) and the change in the body’s mean outline. Finally, by systematic analysis of the error patterns, recommendations were derived and applied to 3D body scans of human subjects resulting in a reduction of error by 67% and 84%.

## 1. Introduction

Three-dimensional (3D) scanning systems are widely used in various applications. Preservation of cultural heritage by digitizing and documenting objects such as ancient artifacts and monuments [1,2,3,4], prototyping and reverse engineering in manufacturing [4], and quality control and inspection in aerospace industries [4] are just a few examples where 3D scanners are well established. In anthropometric surveys [5,6,7] and the fashion industry [8,9,10], 3D body scanners are attracting increasing interest, since a wide range of measurements can be collected within a short time span. Also in the medical field [11,12,13], they are used as a tool to obtain measurements for manufacturing made-to-measure prosthesis and prosthetic socks [14,15], gloves [16], and compression garments [17].

While manual measurements with tape measures are considered the gold standard [13,18], 3D body scanners offer better repeatability [10,19], require less time [11,20,21], and allow non-contact measurements [11,12]. Especially in the medical field, it is crucial to achieve a high accuracy [18,21], since, for example, the measurements for individually manufactured textiles in compression therapy have a direct influence on the applied pressure and consequently on the therapeutic efficacy. Therefore, it is recommended to use stationary or handheld scanning systems [22]. While handheld scanners have the advantage of being mobile and less expensive than stationary systems, stationary 3D body scanners are commonly used as ground truth [22] for the comparison of different scanning systems regarding their accuracy in capturing anthropometric measurements and body shapes. The measurement quality of a specific scanning system can then be quantified by comparing its 3D point cloud to the 3D point cloud of the reference system.

The metric of total difference (Error) is typically used when comparing the same anthropometric measurements on the acquired 3D point clouds. The error can be categorized into three components, some of which are interdependent. The first component is the error due to the properties of the 3D body scanner (Error_Scanner_) stemming from the hardware components used for the acquisition of the object’s 3D data as well as the software used to reconstruct and process the 3D point cloud. Second, environmental influences such as lighting conditions [21,22], temperature and humidity [21] are represented by Error_Environment_. The third component is the subject-dependent error, Error_Subject_, which can have an impact due to movement [23], body hair [10], posture and muscle contraction [18], and its surface properties such as reflectance [23]. Error_Environment_ and Error_Subject_ can be reduced and maintained on a constant and reproducible level by using constant lighting conditions [21] and by using a solid object such as a rigid body model [24]. By keeping these two factors constant, the scanner-related error, Error_Scanner_, can be isolated and investigated.

**Figure 1:**
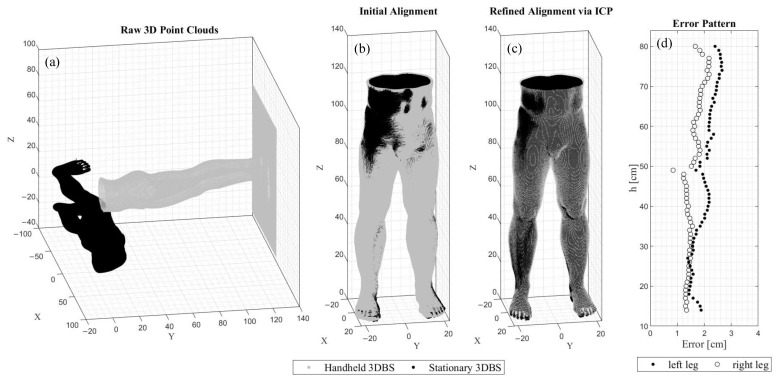
The raw 3D point clouds obtained by a stationary and a handheld 3D body scanner (3DBS) (**a**) in a common coordinate system prior to the alignment, (**b**) after the initial and (**c**) the refined alignment using the ICP algorithm, and (**d**) the resulting error pattern.

In a recent in-house study, 3D point clouds of a rigid body model were acquired using a stationary and a handheld 3D body scanner under controlled lighting conditions. Since the raw 3D point clouds acquired by the different 3D body scanners do use an internal but not a common coordinate system (Figure 1a), they had to be aligned prior to the evaluation of the accuracy. An initial (Figure 1b) and refined alignment (Figure 1c) were performed using the Iterative Closest Point (ICP) algorithm [25,26]. The accuracy of the handheld 3D body scanner was evaluated by calculating the circumferences and comparing them to the circumferences obtained by the ground truth, the stationary 3D scanner. The determined deviations followed a specific error pattern (Figure 1d). It remained unclear whether these errors were due to the performance of the scanner or to a different influencing component such as alignment. Therefore, in this paper it is hypothesized that a fourth component, Error_Alignment_, must be considered when comparing the raw 3D point clouds of a 3D body scanner to those of a different 3D body scanner. 

In the literature, a variety of methods for aligning 3D point clouds are described. Huang et al. [27] provided a comprehensive review of these methods and noted that aligning objects acquired by different 3D sensors is challenging, as outliers and noise, partial overlap, density difference, and scale variations may be present in the point clouds. Furthermore, the combination of current methods being time consuming and having a poor accuracy together with a lack of literature intensifies the problem [27]. In addition, O’Toole et al. [28] demonstrated that alignment between scans of the same source but with slight modifications can result in translational and rotational errors and does not necessarily lead to sufficiently good alignment. Recent publications [29,30,31] address the misalignment problem of 3D point clouds of human bodies by using novel approaches such as deep learning. This can reduce the alignment error, but residual translational and rotational errors are also reported [29,30]. However, these publications intend to reduce the alignment error and evaluate the quality of registration and alignment techniques but do not systematically analyze the effects of alignment errors on the accuracy of measured circumferences of the human lower body in isolation.

Error patterns due to rotational and translational misalignment in 3D body scans of the lower extremities have not been systematically simulated and investigated in detail. Therefore, this paper aims to further enhance the understanding of alignment errors and fill that gap by (1) simulating translational and rotational misalignment and investigating the error focusing on the lower extremities and (2) correlating the error to the body shape. The results derived from the systematic analysis are then applied to further 3D body scans to demonstrate the effect.

## 2. Materials and Methods

### 2.1. 3D Body Scans Used for Simulating the Misalignment

**Figure 2:**
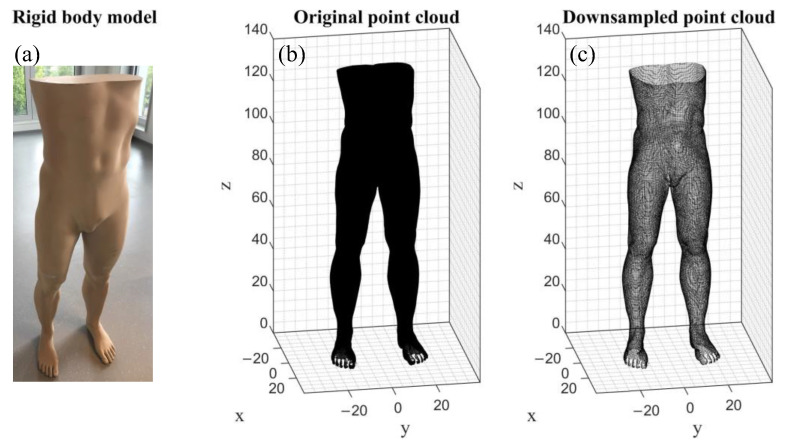
Representation of the body model used for the error simulation. (**a**) Image of the real rigid body model; (**b**) the original point cloud recorded using the VITUS^bodyscan^ with more than 2 million vertices; and (**c**) the same point cloud downsampled to almost 68,000 vertices.

To simulate the misalignment, one raw scan and a copy of it were used to exclude other potential influencing variables such as missing data due to holes and the weighing and distribution of vertices in the 3D point cloud. The scan was acquired using the 3D full body scanner VITUS^bodyscan^ (Vitronic, Dr.-Ing. Stein Bildverarbeitungssysteme GmbH, Wiesbaden, Germany) with a specified accuracy of less than 1 mm [32]. A rigid body model (Figure 2a) derived from a real human body shape of the lower extremities to the trunk was used as the scan object, since objects with a simpler geometry, such as spheres and cylinders, might underestimate the accuracy [23]. It was milled from a polyurethane obomodulan^®^ 500 block, since this material has a dull, non-reflective, homogenous, and smooth surface that is suitable for 3D scanning and can be easily shaped and machined to replicate the exact shape of a real human body. The rigid body model was used to exclude the subject-related influencing parameters and had dimensions of 44.1 cm × 32.2 cm × 136.4 cm (width × depth × height). As the point cloud recorded by the VITUS^bodyscan^ has many redundant vertices to display the 3D body scan (Figure 2b), the scan and its copy were downsampled by 96.8% from 2,116,471 to 68,185 vertices. For this, the numerical software MATLAB R2021b (The MathWorks, Inc., Natick, MA, USA) was used due to the availability of a toolbox for processing 3D point clouds. The pcdownsample function [33] was applied to downsample the 3D point cloud using the grid average downsampling method and a grid box size of 0.5 cm, which merges all points within this box into a single point (Figure 2c). The time required for the simulation and the computation time for calculating the circumferences can be reduced considerably by this step.

Since the VITUS^bodyscan^ is using its own coordinate system, the orientation of the scan axes is changed according to the ISO 20685-1 [34], where the x-axis refers to the sagittal axis, the y-axis to the transverse axis, and the z-axis to the longitudinal axis. The point of origin is the body’s center of gravity translated to the lowest point of the 3D point cloud (z = 0).

### 2.2. Simulation of Misalignment

#### 2.2.1. Translational Misalignment

To simulate the misalignment due to a translational offset, the point cloud of the copied raw scan is successively translated along the x-, y-, and z-axes in positive and negative axial directions. The minimum translation in negative axial direction was chosen to be −1.0 cm and the maximum translation in positive axial direction was chosen to be +1.0 cm. The step size, by which the 3D point cloud is translated iteratively along one of the axes in negative or positive direction, was set to 0.1 cm.

#### 2.2.2. Rotational Misalignment

The rotational misalignment was simulated by rotating the point cloud of the copied raw scan iteratively around one of its principal axes, the x-, y-, and z-axes. The step size was chosen to be 0.1° with the limits of −5.0° in negative rotational direction and +5.0° in positive rotational direction.

#### 2.2.3. Assumptions

**Figure 3:**
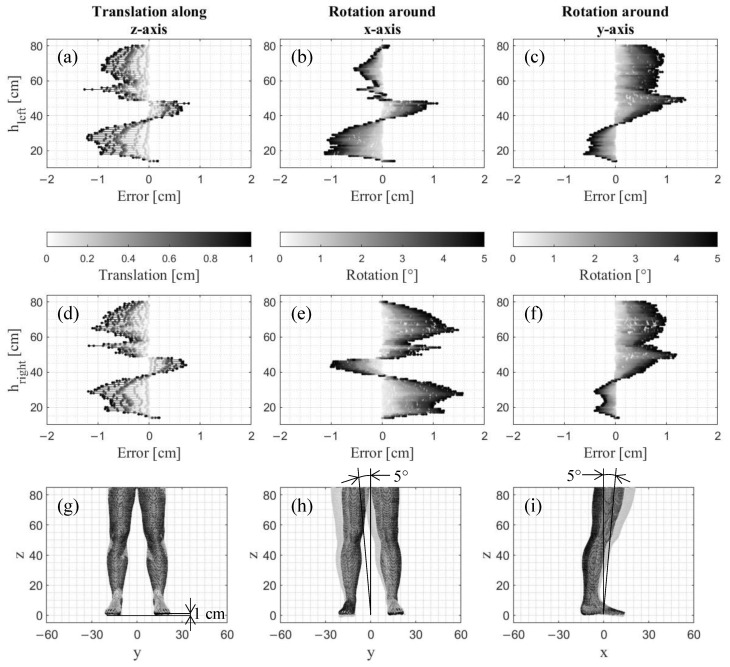
Errors along the height h between 14 cm (ankle) and 80 cm (thigh) in 1 cm steps for (**a**,**d**,**g**) translations along the z-axis, (**b**,**e**,**h**) rotations around the x-axis, and (**c**,**f**,**i**) rotations around the y-axis separately depicted for (**a**–**c**) the left leg and (**d**–**f**) the right leg. The third row (**g**–**i**) shows the 3D point cloud without any translations or rotations (dark grey) and with the maximum translation of 1.0 cm and maximum rotation of 5.0° (light grey).

O’Toole et al. [28] investigated the accuracy of commonly used alignment algorithms on randomly rotated and translated 3D dental scans and found residual rotational errors of 2.52 ± 1.18° and residual translational errors of 139 ± 42 µm. Therefore, it is assumed that the chosen limits of the rotational error of ±5° are reasonable. Considering that the size of a tooth is remarkably smaller than the size of a human body, a limit for the translational offset of ±1.0 cm is considered appropriate when extrapolating the residual translational error of a tooth to the size of a human body. Furthermore, a translation or rotation more than this magnitude is very likely to be detected by visual inspection and thus is to be excluded from an evaluation (Figure 3g–i). However, an alignment error below these values might not be visually detectable and thus could be included as an error without being noticed. The step sizes for translational misalignment and rotational misalignment were chosen to be 0.1 cm and 0.1°, respectively, since an even smaller step size (below 0.1 cm and 0.1°) would only increase the resolution of the error pattern but not change the overall course. A larger step size, on the other hand, would reduce the resolution and might lead to a loss of information. Therefore, the chosen step size is assumed to be adequate.

### 2.3. Circumference Measurement

To measure the circumferences at the required heights of the 3D point cloud, a MATLAB algorithm was developed based on the method reported by Salleh et al. [35]. The algorithm processes the vertices of the 3D point cloud and the height at which the circumference is to be determined. As a result, the 3D point cloud is sliced at the specified height and all vertices are projected onto a 2D plane parallel to the x–y plane. To ensure that the number of vertices is sufficient to calculate the circumference, the vertices within 0.5 cm above and below the target height are included in the 2D plane. Hence, all vertices of the 3D point cloud within a slice thickness of 1.0 cm, which corresponds to the width range of a standard tape measure for anthropometric body measurements as recommended by ISO 8559-1 [36], are used to calculate the circumference at the specific target height. Within this projection, the circumference is calculated using a moving average through the projected vertices.

A comparison to the proprietary anthropometric software Anthroscan 3.5.1 that uses a total slice thickness of 4 mm (Avalution GmbH, Kaiserslautern, Germany) shows no significantly different circumference values using the developed MATLAB algorithm (t(133) = 5.987, *p* < 0.001). Therefore, the MATLAB algorithm can be used to determine the circumferences from a 3D point cloud.

### 2.4. Evaluation of the Measurement Error

To compute the circumferences, the 3D point clouds were sliced along the x–y plane starting at a height of 14 cm which is the smallest circumference of the leg and represents the ankle up to a height of 80 cm, representing the gluteal fold. The height range between the ankle and the gluteal fold was chosen due to its relevance to manufacturing medical compression garments for the lower extremities as defined by RAL-GZ 387/1 [37]. The step size was set to 1.0 cm, again being in the same width range of a standard tape measure recommended by ISO 8559-1 [36]. The error was defined as the difference between the measured value and the reference value [38]. In this study, the reference value refers to the circumference of the non-translated and non-rotated 3D point cloud (C_Ref_), and the measured value refers to the circumferences of the transformed 3D point cloud (C_Trans_) used to simulate the misalignment. Both circumferences are reported in cm. The total error due to misalignment (Error_Total_) is calculated as follows:

(1) $${Error}_{Total}=C_{Trans}-C_{Ref}\;[cm]$$

### 2.5. Verification

By systematic analysis of the simulated misalignment and the resulting error patterns, recommendations were derived to find landmarks that are most robust to misalignment (see Section 3.1.3). Since these recommendations were derived from simulations using the rigid body model, they must be applied to a variety of body shapes to verify their applicability. Therefore, a dataset of *n* = 30 subjects (n_female_: 15, n_male_: 15, age: 39 ± 13.2 years, BMI: 26.2 ± 4.3 kg/m^2^) was used, which contains the 3D point clouds of their lower extremities that were acquired using the VITUS^bodyscan^. This dataset was generated as part of a previous study conducted in accordance with the Declaration of Helsinki [39]. All subjects were informed prior to the start of the study and signed a written informed consent.

Similar to the simulated misalignment of the 3D point cloud of the rigid body model, the 3D point clouds of the subjects were first downsampled. Since misalignment can be composed of a combination of rotational and translational errors, a combined misalignment was used based on the values reported in the literature for residual rotational errors of 0.56 ± 0.38° [20] or 1.7° [29] and residual translational errors of 0.72 cm [29]. Therefore, a combined misalignment of +0.5 cm translation along the z-axis, +1.0° around the y-axis, and +1.0° around the x-axis was applied to the 3D point clouds in this verification. The derived recommendations were then applied to the 3D point clouds and Error_Total_ was calculated according to Equation (1) at the recommended landmarks. Likewise, Error_Total_ was calculated at the knee, which is a prominent and easily identifiable landmark in healthy subjects and is defined in ISO 8559-1 [29]. Since the two landmarks have different circumferences, they must be compared relatively to determine if the recommended landmark is more robust to misalignment. Therefore, a comparison between the error at the recommended landmark and the error at the knee was made by using the absolute percentage error (APE) for each subject and the mean absolute percentage error (MAPE) for the whole *n* = 30 subjects according to the formulas below:

(2) $$\mathrm{APE}=\left|\frac{C_{Trans}-C_{Ref}}{C_{Ref}}\right|\times 100\;[\%]$$

(3) $$\mathrm{MAPE}=\frac{1}{n}\sum_{m=1}^{n}\left|\frac{C_{Trans,m}-C_{Ref,m}}{C_{Ref,m}}\right|\times 100\;[\%]$$

## 3. Results and Discussion

**Figure A1:**
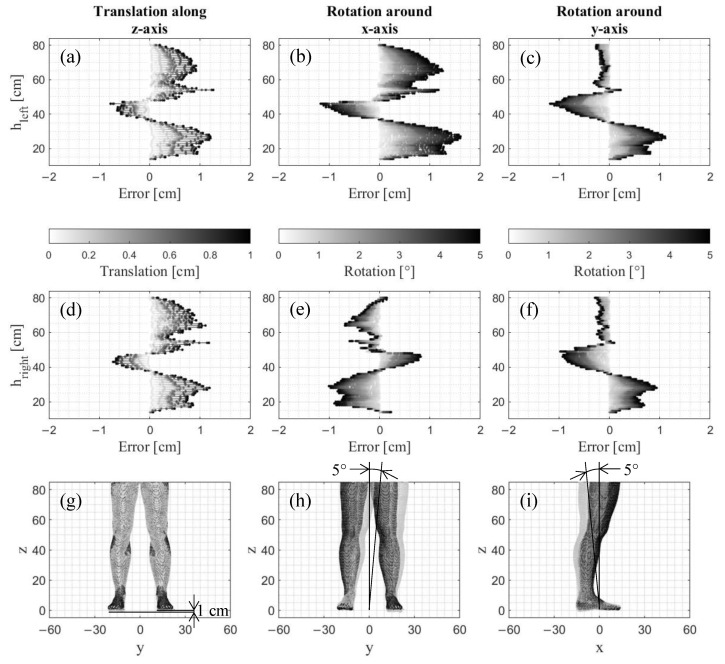
Errors along the height h between 14 cm (ankle) and 80 cm (thigh) in 1 cm steps for (**a**,**d**,**g**) negative translations along the z-axis, (**b**,**e**,**h**) negative rotations around the x-axis and (**c**,**f**,**i**) negative rotations around the y-axis separately depicted for (**a**–**c**) the left and (**d**–**f**) the right leg. The third row, (**g**–**i**), shows the 3D point cloud without any translations or rotations (dark grey) and with the maximum negative translation of 1 cm and maximum negative rotation of 5° (light grey).

The results are presented only for translations in positive axial direction and for rotations in positive direction of rotation to preserve a clear overview. However, the results found here are applicable to the negative translations and rotations as well. In the Appendix A, Figure A1 shows the course of the errors for a translation along the z-axis and the rotations around the x-axis and y-axis in negative direction.

### 3.1. Translational and Rotational Misalignment Error

No errors occurred neither for a translation along the x-axis or y-axis nor for a rotation around the z-axis, as the MATLAB algorithm slices the point cloud along the x–y plane. As a translation along this plane and a rotation around an axis perpendicular to this plane has no influence on the error, they only appear for a translation along the z-axis as well as for rotations around the x-axis and y-axis (Figure 3).

In general, the more the point cloud is translated along the z-axis, the higher the magnitude of Error_Total_ (Figure 3a,d). The same applies to the errors caused by the rotation around the x-axis (Figure 3b,e) and the y-axis (Figure 3c,f). This demonstrates a clear trend indicating that the errors can be extrapolated for larger angles of rotation and translational offsets. The maximum absolute errors ranged from 1.19 cm for translation along the z-axis to 1.36 cm for the rotation around the y-axis and 1.57 cm for the rotation around the x-axis. The absolute values are valid specifically for this body model and may differ for other body shapes.

#### 3.1.1. Translational Component of the Misalignment Error

**Figure 4:**
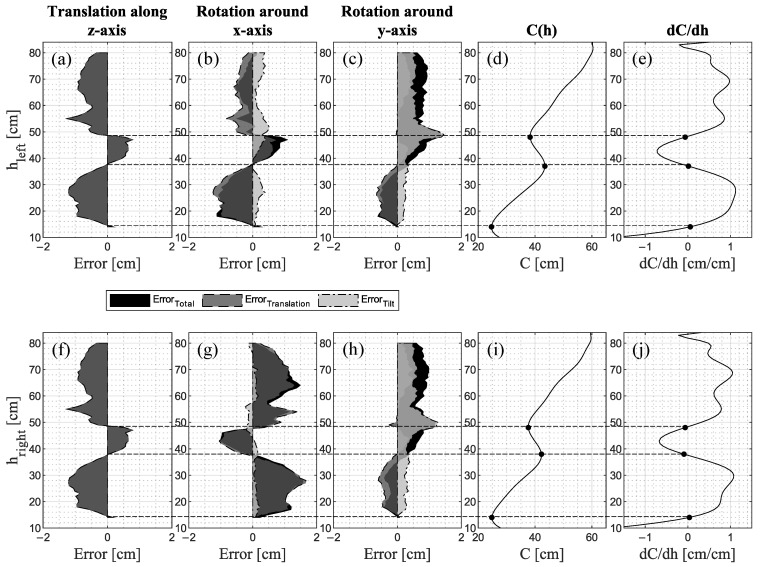
(**a**–**c**,**f**–**h**) Errors divided according to their translational and tilt components, which in turn add up to the total error combined with (**d**,**i**) the circumferential course C(h) and (**e**,**j**) the first derivative representing the change of the circumference dC/dh.

As shown in Figure 4, Error_Total_ is a superposition of a translational (Error_Translation_) and a tilt (Error_Tilt_) component. The latter is explained in Section 3.1.2. The errors caused by a translation along the z-axis (Figure 4a,f) occur solely due to the translational offset and therefore have no tilt component.

**Figure 5:**
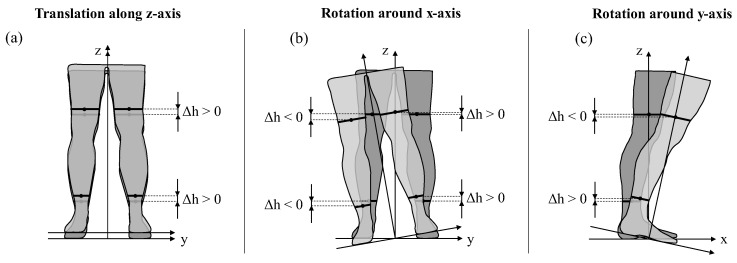
Schematic illustration of the reference 3D point cloud (dark grey) and the translated 3D point cloud (light grey) to represent the height difference Δh for (**a**) translation along the z-axis, (**b**) rotation around the x-axis, and (**c**) rotation around the y-axis.

By translating along the z-axis (Figure 4a,f), Error_Translation_ becomes negative in regions of increasing circumference (Figure 4d,i) since, according to Equation (1), the translated 3D point cloud is compared to the non-translated 3D point cloud at the same height distance from the ground (z = 0). Hence, by translating along the z-axis in positive direction, a smaller circumference at the translated 3D point cloud is compared to a larger circumference at the reference 3D point cloud, resulting in a negative error. Conversely, Error_Translation_ becomes positive in regions of decreasing circumferences (Figure 4d,i). At distinctive locations where the change of circumference dC/dh (Figure 4e,j) approaches 0, a change of sign occurs for Error_Translation_. These locations correspond to the maximum and the minima of the leg’s circumferences, representing the anatomical locations of the ankle (both legs: 14 cm), the mid-calf (left leg: 37 cm, right leg: 38 cm), and the lower patella region (both legs: 48 cm). The course of Error_Translation_ due to rotation around the x-axis has similarities to Error_Translation_ due to translation along the z-axis (Figure 4b) but differs in magnitude and sign for the right leg (Figure 4g). The same applies to the rotation around the y-axis (Figure 4c,h) up to a height of 52 cm, above which Error_Translation_ becomes positive. The reason for it is the height difference Δh = h_Transformed_ − h_Reference_ between the points where the transformed 3D point cloud and the non-transformed reference 3D point cloud are sliced. Figure 5 illustrates the height difference Δh and indicates the associated sign.

**Figure 6:**
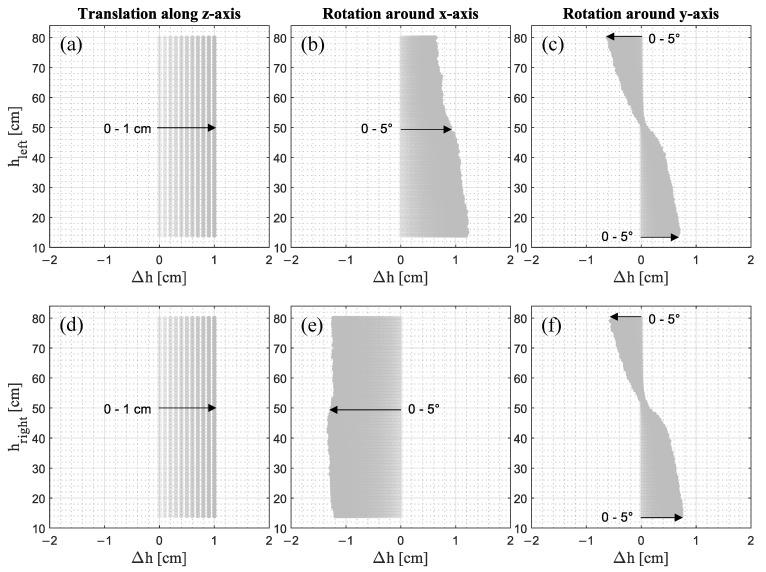
Difference of heights Δh between the height where the translated and rotated point cloud is sliced and the height of the reference point cloud for (**a**,**d**) translations between 0 and 1.0 cm and rotations between 0° and 5.0° around (**b**,**e**) the x-axis and (**c**,**f**) y-axis.

Since the point cloud is translated steadily in 0.1 cm steps along the z-axis, Δh is constant along the height for the translation along the z-axis (Figure 6a,d). By contrast, for rotation around the x-axis, Δh is positive with a decreasing tendency as the height increases for the left leg (Figure 6b), and negative with a nearly constant tendency for the right leg (Figure 6e). The positive sign results from the upward movement of the left leg due to the rotation around the x-axis, while the negative sign is due to the simultaneous downward movement of the right leg, as can be seen in Figure 5b. The tendencies occur due to the distance between the points where the point cloud is sliced and the coordinate origin, representing the center of rotation. Since the rotation occurs on a circular path of different radii, a farther distance in the vertical direction leads to a smaller offset in the z-direction. A farther distance in the horizontal direction leads to a larger offset in the z-direction. Consequently, Δh is also smaller or larger, respectively. Additionally, since the body model is derived from a human being and thus there is no perfect symmetry to the sagittal plane, the origin is not exactly in the center of the legs but may be offset and therefore result in a different tendency of Δh for the left and right leg. The same applies to the rotation around the y-axis (Figure 6c,f) where for both the left and right leg, Δh is positive for the leg section up to 53 cm, whereas the section above is negative. Thus, the change of signs characterizes the point where the translation of the point cloud changes from up to downwards.

**Figure 7:**
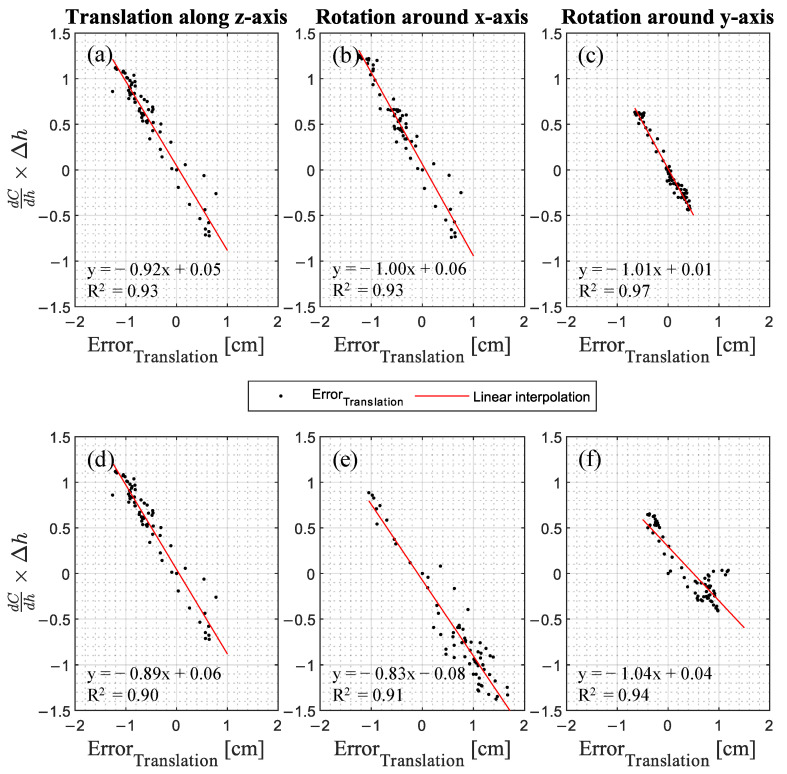
The change of circumference dC/dh multiplied by the difference of heights Δh versus the Error_Translation_ for (**a**,**d**) the translation along the z-axis, (**b**,**e**) the rotation around the x-axis, and (**c**,**f**) the y-axis presented for (**a**–**c**) the left and (**d**–**f**) right leg.

To investigate the interrelationship, the product of the translational component and differential change of the circumferences per change of height of the non-translated or non-rotated point cloud versus Error_Translation_ is shown in Figure 7.

For all translational components of the errors in dependence of the change of circumference dC/dh multiplied by the difference of height Δh, a strong correlation exists (R^2^ = 0.90, … 0.97). Therefore, it can be concluded that the larger the product of the change of circumference and the height difference, positive or negative, the more pronounced is Error_Translation_. Since the change of circumference is an abstraction of the leg shape, it can be inferred that Error_Translation_ occurs shape-dependently.

#### 3.1.2. Tilt Component of the Misalignment Error

**Figure 8:**
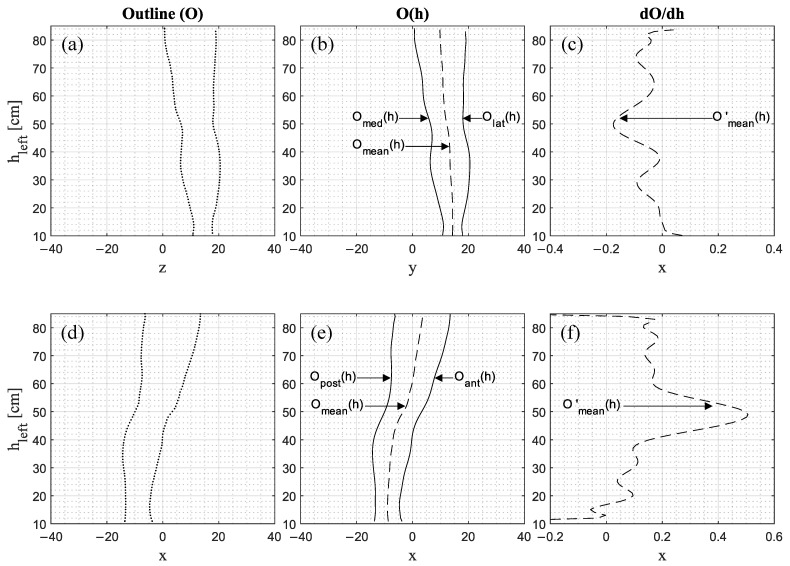
The (**a**) frontal and (**d**) lateral outline of the left leg along the height h. The outline functions on the (**b**) medial O_med_ and lateral side O_lat_ as well as on the (**e**) posterior O_post_ and anterior side O_ant_ of the left leg, including their mean outline function O_mean_. The third column (**c**,**f**) shows the derivative of the mean outline function O’_mean_.

In addition to Error_Translation_, Error_Tilt_ occurs due to the rotation of the 3D point cloud around the x-axis and y-axis, since the sectional plane is no longer parallel to the x–y plane but tilted by the angle of rotation. By this, the body part is intersected in another angle, resulting in larger or smaller cross-sectional slices—depending on the leg shape at the respective position. As shown in Figure 8a,d, the points on each centimeter of the outline are used to calculate the medial O_med_(h) and lateral O_lat_(h) outline function (Figure 8b) as well as the posterior O_post_(h) and anterior O_ant_(h) outline function (Figure 8e). The mean outline function O_mean_(h) is calculated based on the leg outlines and its corresponding derivative is used as the change in the mean outline function O’_mean_(h) = dO_mean_/dh (Figure 8c,f).

**Figure 9:**
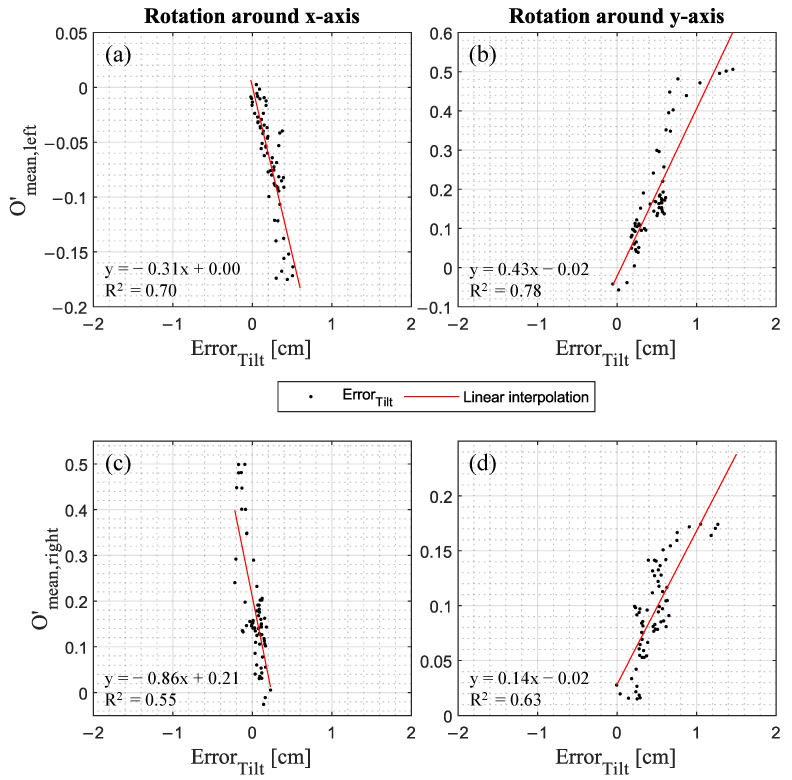
The change of the mean outlines O’_mean_ versus the Error_Tilt_ for (**a**,**c**) the rotation around the x-axis and (**b**,**d**) y-axis presented for the (**a**,**b**) left leg and (**c**,**d**) right leg, respectively.

As Figure 9 indicates, correlations exist between Error_Tilt_ and the change of the mean outline functions O’_mean_(h) ranging from R^2^ = 0.55 to R^2^ = 0.78. Thus, the leg shape has an influence on the tilt component of the error and consequently on Error_Total_, considering that the change of the mean outline function is derived by a 2D projection, whereas the Error_Tilt_ is calculated based on the 3D point cloud vertices.

#### 3.1.3. Application of the Results

By analyzing the error patterns, correlations between the change of circumferences along the height multiplied by the difference of the heights and Error_Translation_ as well as between the change of the legs’ mean outline and Error_Tilt_ were shown. Considering these results, two recommendations can be formulated to select the most robust landmarks as references for comparing 3D point clouds from different 3D body scanners.

- Recommendation 1:
  Calculate the change of the circumference along the height as well as the change of the reference point clouds’ mean outline and use the height where both approach 0 as a landmark to compare the 3D point clouds.

- Recommendation 2:
  Use the ankle or calf as landmarks to compare the 3D point clouds. Since the ankle or calf are usually the landmarks where the local circumference is minimum or maximum, respectively, the change of the circumference at these landmarks approaches 0, making them more robust landmarks.

### 3.2. Verification of the Results

Table 1 summarizes the APE at the recommended landmark and at the knee and indicates the corresponding heights for the left and the right leg of the 3D point clouds for each of the 30 subjects. In addition, the difference between the APE at the recommended landmark and APE at the knee is reported to indicate a decrease (−) or increase (+) in error. The MAPE and the mean difference are indicated in the penultimate row and their corresponding one standard deviation (STD) in the last row.

The MAPE ± STD at the recommended landmark was 0.31 ± 0.24% for the left leg and 0.13 ± 0.08% for the right leg, whereas the MAPE at the knee was 0.94 ± 0.42% and 0.82 ± 0.37% for the left and right leg, respectively. Thus, the MAPE was reduced by 0.63 pp for the left leg and by 0.69 pp for the right leg. As the MAPE was reduced from 0.94% to 0.31% for the left leg and from 0.82% to 0.13% for the right leg, it can be concluded that using the landmarks recommended by following Recommendation 1 instead of a prominent landmark such as the knee, the MAPE was reduced by 67% and 84% for the left and right leg, respectively.

In 9 out of 30 cases, the height of the recommended landmarks on the left leg were at the ankle and in 21 out of 30 cases at the calf. Similarly, 6 out of 30 recommended landmarks were at the ankle of the right leg and 21 out of 30 were at the calf. For three cases on the right leg of subjects 12, 14, and 15, a position above knee level is recommended. This supports Recommendation 2 described in Section 3.1.3, as a total of 57 out of 60 landmarks were either in the ankle or calf region.

### 3.3. Limitations

The limitations of this paper include aspects of the methodology used to simulate the misalignment and the application of the derived recommendations.

First, a range of ±5.0° and ±1.0 cm with a step size of 0.1° and 0.1 cm, respectively, was used to simulate the misalignment, since a misalignment higher than these values is likely to be detected during visual inspection and the resolution was assumed to be adequate. The limits were derived from residual errors published in a paper on the accuracy of commonly used alignment algorithms for 3D dental scans. In practice, larger rotational and translational errors may occur that would not be detected in an automated process without a visual inspection step. Second, the slice thickness was chosen to be 1.0 cm, which means that all points below and above 0.5 cm of the target height are included to calculate the circumference. This thickness was chosen, since it corresponds to the width range of a standard tape measure recommended by ISO 8559-1 [36]. The slice thickness may influence the measured circumferences, especially for the rotated 3D point cloud, and thus the calculated errors. Third, a rigid body model was used as an object to simulate the misalignment to exclude subject-related influencing factors such as movement, posture, body hair, and muscle contraction. Therefore, the absolute values of the errors presented in Section 3.1. are only valid for this specific body model. Fourth, since the region of interest was the lower extremities between the ankle and the thigh, other regions are not considered in this paper and the recommendations are limited to this height.

The recommendations were applied to further 3D point clouds to verify their generalizability. These 3D point clouds stemmed from the lower extremities of 30 healthy human subjects with a distinct body shape and clearly visible ankle and calf regions. Hence, the recommendations are valid for a similar body shape to that represented by the rigid 3D body model. Furthermore, a combined misalignment value was chosen based on the residual errors reported in the literature. However, in a real-world scenario, a variety of misalignment combinations may occur with rotational and translational degrees that likely differ from those used in this paper. The results obtained in Section 3.2 show reduced MAPE values when using the recommended landmark instead of a prominent landmark. Since to the authors’ best knowledge, there is no known literature available on analyzing error patterns in rigid 3D body scans and using the derived recommendations to suggest landmarks that are more robust to alignment errors, a comparison between the results in this paper and the literature is not feasible.

## 4. Conclusions

This paper aimed to enhance the understanding of alignment errors on the accuracy of measured circumferences. Therefore, (1) two different types of misalignments, translational and rotational, were simulated by using the 3D point cloud of a rigid body model in comparison to its copy. It was demonstrated that the occurring total error consists of a translational and a tilt component. (2) The translational component of the error is due to the change of circumferences along the height multiplied by the difference of height, while the tilt component is due to the change in the legs’ mean outline. Both error components are therefore strongly dependent on the shape of the scanned body if a misalignment is present. Additionally, two recommendations derived from the correlations were provided to select the most robust landmarks by either using the landmark where the change of the circumference and the change of the legs’ mean outline functions approach 0 or by using the ankle or calf as landmarks to compare the 3D point clouds. Furthermore, by applying the recommendations to 3D body scans of real human subjects, it was shown that the MAPE was reduced by 67% and 84% for the left and right legs, respectively. Considering the lack of literature that analyzes error patterns and recommends robust lower extremity landmarks for the comparison of anthropometric measurements of two 3D body scanners, the results found in this paper can be considered as a cornerstone for 3D body scans. Finally, it can be concluded that Error_Alignment_ must be considered when comparing the raw 3D point clouds of a 3D body scanner to those of a different 3D body scanner. By addressing the alignment errors in this paper, researchers, practitioners, and industry professionals can make more informed decisions in selecting and evaluating the 3D scanning system of interest for their application area, such as anthropometric surveys, the fashion industry, and the medical field.

Further analysis should investigate to what extent the total slice thickness affects the errors, as the points above and below the target height are included in the calculation of the circumferences and thus have an influence on their value. Therefore, a total slice thickness between 0.5 and 1.0 cm should be analyzed corresponding to the width range of a standard tape measure recommended by ISO 8559-1 [36]. Moreover, the reduction of error for further translational and rotational combinations of misalignment should be explored, as in this paper a fixed misalignment was used to verify the recommendations although an occurring misalignment can be composed of a variety of combinations.

## Figures and Tables

**Table 1:** The heights of the recommended landmark and the knee with the absolute percentage errors (APE) and the difference between their values (Diff.) as well as the mean value and one standard deviation (STD) for the left and right legs of all 30 subjects. All values are rounded to the second decimal place.

	Left Leg					Right Leg				
	Height (cm)		APE (%)		Diff. (pp)	Height (cm)		APE (%)		Diff. (pp)
Subject	Recommended	Knee	Recommended	Knee		Recommended	Knee	Recommended	Knee	
1	35	44	0.45	1.30	−0.85	34	45	0.15	1.10	−0.95
2	41	58	0.02	0.65	−0.63	40	57	0.22	0.47	−0.25
3	11	47	0.39	0.58	−0.19	13	47	0.27	0.37	−0.09
4	31	44	0.10	1.05	−0.95	31	42	0.11	1.07	−0.96
5	31	42	0.20	0.47	−0.27	32	41	0.08	0.47	−0.40
6	36	51	0.21	0.27	−0.06	36	52	0.15	0.82	−0.67
7	37	47	0.25	0.46	−0.21	36	47	0.03	0.53	−0.51
8	11	45	0.31	0.89	−0.58	11	45	0.24	1.11	−0.87
9	32	42	0.56	0.17	+0.39	33	44	0.08	0.22	−0.14
10	37	50	0.24	0.89	−0.65	38	51	0.06	0.57	−0.51
11	39	55	0.29	0.98	−0.69	14	55	0.09	0.80	−0.71
12	39	55	0.36	0.60	−0.23	60	58	0.10	0.28	−0.18
13	12	44	0.33	0.90	−0.57	30	43	0.16	1.05	−0.89
14	13	53	1.06	0.22	+0.84	58	53	0.03	0.37	−0.34
15	32	45	0.30	1.42	−1.12	49	43	0.19	1.38	−1.19
16	36	48	0.36	0.55	−0.20	37	50	0.08	0.75	−0.67
17	36	50	0.48	1.08	−0.60	36	49	0.02	0.87	−0.84
18	12	48	0.71	1.27	−0.56	34	47	0.19	0.75	−0.55
19	36	49	0.31	1.34	−1.04	35	50	0.01	0.48	−0.47
20	34	46	0.21	1.09	−0.88	14	46	0.13	0.64	−0.52
21	36	48	0.09	1.16	−1.07	36	48	0.28	0.91	−0.63
22	35	47	0.28	1.48	−1.19	13	46	0.31	0.96	−0.65
23	38	53	0.42	1.62	−1.21	15	52	0.13	1.12	−0.99
24	15	51	0.27	0.78	−0.51	34	50	0.06	0.69	−0.62
25	12	46	0.07	1.31	−1.24	34	45	0.26	2.09	−1.83
26	33	47	0.02	1.57	−1.55	33	47	0.02	0.97	−0.96
27	32	46	0.04	1.19	−1.15	31	44	0.09	1.29	−1.20
28	15	55	0.10	0.38	−0.29	39	54	0.03	0.52	−0.49
29	13	45	0.88	1.30	−0.43	32	42	0.13	1.00	−0.87
30	34	49	0.14	1.29	−1.15	34	47	0.09	1.00	−0.91
Mean			0.31	0.94	−0.63			0.13	0.82	−0.69
STD			0.24	0.42	0.51			0.08	0.37	0.35

## Data Availability

Data are contained within the article.

## Appendix A

In Figure A1, the errors along the height are displayed for negative translations along the z-axis and negative rotations around the x-axis and y-axis.

The course of the errors for translation in positive z-direction shown in Figure 3 is the same as the course of error in negative translational direction, but with a reversed sign. The course of error for the rotation around the x-axis in negative direction corresponds to the rotation around the x-axis in positive direction with the difference that the courses of error of the right and the left leg are interchanged. Note that slight differences are present since the rigid body model is not symmetrical and differs in the circumferences for the left and right leg. For the rotation around the y-axis, the course of the errors is similar to the course displayed in Figure 4c,f but differing in the magnitude. This becomes most apparent at heights above the knee. Since a negative rotation around the y-axis leads to a consistently negative Δh with a decreasing tendency, Error_Translation_ approaches 0 whereas Error_Tilt_ remains positive. Therefore, the error is negative with a lower magnitude.
